# Antithrombotic Therapy in Patients with Coronary Artery Disease and Prior Stroke

**DOI:** 10.3390/jcm10091923

**Published:** 2021-04-29

**Authors:** Elisa Bellettini, Leonardo De Luca

**Affiliations:** Department of Cardiosciences, U.O.C. of Cardiology, Azienda Ospedaliera San Camillo-Forlanini, 00152 Roma, Italy; bellettini.elisa@gmail.com

**Keywords:** antithrombotic therapy, clopidogrel, ticagrelor, prasugrel, aspirin, rivaroxaban, acute coronary syndromes, prior stroke

## Abstract

Patients with coronary artery disease (CAD) and prior cerebrovascular events (CVE) are frequently faced in clinical practice and present a high rate of both ischemic and bleeding events. For these reasons, the antithrombotic management is particularly challenging in this subgroup of patients. Recent trials suggest that, although a potent antiplatelet strategy is safe in the acute phases of myocardial ischemia for these patients, the risk of major bleeding complications, including intracranial hemorrhage, is extremely high when the antithrombotic therapy is prolonged for a long period of time. Therefore, especially in patients with chronic CAD and history of CVE, the antithrombotic management should be carefully balanced between ischemic and bleeding risks. The present review is aimed at critically evaluating the available evidence to help make these crucial clinical decisions regarding the better antithrombotic therapy to use in this high-risk subgroup of patients.

## 1. Introduction

Patients with ischemic stroke present a 4-fold increased risk for coronary artery disease (CAD) compared to patients without cerebrovascular diseases (CVD) [1]. Patients with both CAD and CVD present a 3 times higher risk for stroke and intracranial bleeding compared with patients without history of CVD [1,2,3,4,5]. For these reasons, literature agrees in identifying this group of patients as particularly challenging to manage, especially when the right balance between safety and efficacy of antithrombotic treatment needs to be find.

## 2. Prevalence of Stroke in Patients with CAD

Patients affected by CAD rarely report a history of stroke or transient ischaemic attack (TIA). In the Global Registry of Acute Coronary Events (GRACE) Registry, 8% of the overall population with acute coronary syndromes (ACS) enrolled reported a prior CVD [2]. Similar rates of CVD have been detected in recent nationwide registries on consecutive ACS patients and in contemporary clinical trials including patients with ACS or stable CAD [3,4,5] (Table 1 and Table 2). Likewise, in a cohort of more than 26.000 patients with CAD included in the REACH (REduction of Atherothrombosis for Continued Health) registry, the prevalence of previous CVD was approximately 17% [6]. 

Patients with CAD and CVD are generally older, more likely to have history of angina, myocardial infarction (MI), heart failure, coronary artery bypass surgery, diabetes, hypertension, dyslipidaemia, and atrial fibrillation [2,3,6]. 

## 3. Prior Stroke and Ischemic Risk

Notably, the coexistence of both CAD and CVD denotes a higher and more diffuse atherosclerotic burden [7]. In the GRACE Registry [2], the presence of prior stroke or TIA was associated with a double risk of in-hospital and 6-month all-cause mortality. Likewise, the risk of MI and major cardiovascular events (MACE) at 6-months was increased, even after multivariable adjustment for baseline differences. Accordingly, previous stroke resulted as an independent predictor of six-month mortality and MACE [2]. In the REACH registry, patients with both CAD and CVD history reported a higher rate of all-cause mortality (17.8% versus 11.2%, *p* < 0.001), mainly due to a major rate of cardiovascular death (12.2% vs. 7.1%, *p* < 0.001) and cardiovascular events (24.9% versus 13.3%, *p* < 0.001) [6]. Notably, in the sensitivity analysis, patients with prior stroke showed an almost 4-fold increased risk of recurrent stroke. The global ischemic risk in this subgroup remained high even after adjustment for baseline risk, with an estimated HR of 1.52 for the combined risk of cardiovascular (CV) death, MI, or stroke [6]. 

## 4. Prior Stroke and Risk of Bleeding 

As history of CVD increases the ischemic risk, it also generates bleeding concerns. In general, the coexistence of CAD and history of CVD is associated with a high bleeding rate, both for non-fatal hemorrhagic stroke as for bleeding requiring hospitalization and transfusion [6]. Notably, the risk of non-fatal hemorrhagic stroke is particularly high in patients receiving dual antiplatelet therapy (DAPT) [6]. 

## 5. Antithrombotic Therapy: A Challenging Issue

The concomitance of high ischemic and bleeding risk makes the choice of the correct antithrombotic regimen for these patients particularly challenging. 

The present review is aimed at critically evaluating the available evidence on antithrombotic therapies in patients in sinus rhythm with acute or chronic CAD and history of CVD, without considering the antithrombotic strategies tested in the acute phase of cerebrovascular accidents.

A literature search of Medline, Excerpta Medica Database (EMBASE), and Google Scholar was conducted for published articles from database inception to February 2021. Examples of the terms used in the search strategy included ‘stroke, ‘coronary artery disease’, ‘acute coronary syndromes’, ‘antiplatelet therapy’, ‘aspirin’, ‘clopidogrel’, ‘ticlopidine’, ‘ticagrelor’, ‘dual antiplatelet therapy’, ‘oral anticoagulation therapy’, ‘warfarin’, ‘rivaroxaban’, ‘revascularization’, ‘benefits’, ‘mortality reduction’, and relevant individual risk factors. Identified citations were considered for inclusion by the first author. Full-text versions of relevant abstracts were obtained for inclusion and summarized qualitatively. 

### 5.1. Antiplatelet Therapy in ACS 

The concerns in balancing ischemic and hemorrhagic risk in this high-risk group of patients are even amplified in the case of ACS. Nowadays, DAPT, composed by the association of a P2Y12 receptor antagonist (e.g. clopidogrel, ticagrelor or prasugrel) with aspirin (ASA), is the standard of care for patients with ACS [8,9,10,11,12].

The CURE (Clopidogrel in Unstable Angina to Prevent Recurrent Events) trial firstly demonstrated the efficacy of DAPT, specifically clopidogrel added to ASA, in patients presenting with non-ST-segment elevation ACS (4% with history of prior stroke). DAPT reduced the incidence of MACE (HR 0.80, 95% CI 0.72–0.90; *p* < 0.001) and other ischemic endpoints compared to ASA alone [13]. DAPT also registered a trend toward fewer ischemic strokes, without increase in the rate of hemorrhagic stroke. Regarding safety, major bleeding in the DAPT group resulted significantly higher compared to ASA alone (3.7% vs. 2.7%; *p* = 0.001), except for life-threatening bleeding (2.1% vs. 1.8%; *p* = 0.13) [13,14]. 

The PLATO (Platelet Inhibition and Patient Outcome) trial demonstrated the superiority of ticagrelor, a potent, reversibly binding, direct-acting, P2Y12 receptor antagonist, over clopidogrel, when added to ASA for 1 year after an ACS [8]. Ticagrelor 90 mg bid reduced the primary composite end point, a composite of CV death, MI, and stroke, without increasing the overall rate major bleedings [8]. In the subgroup of 1152 patients (6.2%) with a history of CVD, a higher rate of primary composite endpoint (19.9% versus 10.1%), CV death (9.7% versus 4.2%), MI (11.5% versus 6.0%), death from any causes (10.5% versus 4.9%), stroke (3.4% versus 1.2%), and intracranial bleeding (0.8% versus 0.2%) was reported compared to those without prior stroke or TIA [15]. In this subgroup, ticagrelor consistently reduced the primary composite outcome (HR 0.87, 95% CI 0.66–1.13; interaction *p* = 0.84) and total mortality (HR 0.62; 95% CI 0.42–0.91) at 1 year. The rate of bleedings was similar in both arms, and intracranial hemorrhage (ICH) was rare [16]. Therefore, ticagrelor, when used in patients with ACS and history of CVD, despite its more potent antithrombotic effect, has been demonstrated to reduce the incidence of ischemic events without a significant increase in hemorrhagic complications, leading to a favorable net clinical benefit compared to clopidogrel. 

In the TRITON-TIMI-38 (Trial to Assess Improvement in Therapeutic Outcomes by Optimizing Platelet Inhibition with Prasugrel–Thrombolysis in Myocardial Infarction-38) trial, prasugrel, another potent P2Y12 inhibitor, demonstrated its superiority over clopidogrel in ACS patients undergoing percutaneous coronary intervention (PCI) [9]. The primary efficacy end point was significantly reduced by prasugrel (HR 0.81, 95% CI 0.73–0.90; *p* < 0.001), mainly due to spontaneous and peri-procedural MI reduction (HR 0.76, 95% CI 0.67–0.85; *p* < 0.001), with no differences in terms of stroke or mortality compared to clopidogrel. In addition, prasugrel increased the rate of TIMI major bleedings by 32%, with a higher rate of life-threatening bleeding (HR 1.52, 95% CI 1.08–2.13) and fatal major hemorrhages (0.4% versus 0.1%, HR 4.19, 95% CI 1.58–11.11; *p* = 0.002) [9]. However, the composite of death from any cause, nonfatal MI, stroke, and TIMI major hemorrhage favored prasugrel over clopidogrel (HR 0.87, 95% CI 0.79–0.95) [9]. Notably, in the subgroup of patients with history of CVD, there was no difference between prasugrel and clopidogrel in the rate of primary efficacy end point (HR 1.37, 95% CI 0.89–2.13; *p* = 0.15,) with a trend toward a greater rate of TIMI major bleeding (*p* = 0.06), including ICH (*p* = 0.02) in the prasugrel group [9]. Hence, prasugrel can be potentially harmful if used in case of previous CVD, and it is therefore contraindicated by main regulatory authorities and international guidelines in this subset of patients [11,12,17]. 

Recently, the CHAMPION PHOENIX (A Clinical Trial Comparing Cangrelor to Clopidogrel Standard Therapy in Subjects Who Require Percutaneous Coronary Intervention) trial evaluated cangrelor, a potent, intravenous, ADP receptor antagonist, in patients undergoing elective or urgent PCI [18]. Patients were randomly assigned to receive a bolus and infusion of cangrelor or a loading dose of clopidogrel. The odds of an ischemic event were 22% lower with cangrelor compared to clopidogrel, without any significant increase in bleeding complications (*p* = 0.44). In the 5% of the total population with a history of CVD, the reduction in primary end point was consistent with the overall population (interaction *p* = 0.97) as across multiple prespecified subgroups, without a significant increase in bleeding (interaction *p* = 0.54) [18]. Therefore, cangrelor can represent a valid option for patients presenting with ACS and history of CVD, due to its safety profile and net clinical benefit confirmed in this specific subgroup of patients. 

### 5.2. Anticoagulant Drugs in Patients with Recent ACS and Sinus Rhythm

After an ACS, an excess in thrombin generation may persist for a long time after the acute phase [19]. This persisting hyper-coagulable state could partially explain the high incidence of recurrent CV events that occurs in these patients, despite standard medical therapy. 

Few studies initially suggested that an anticoagulant treatment with vitamin K antagonists was able to improve CV outcome, but it was also associated with an increased risk of major and life-threatening bleeding events compared to placebo [19]. Subsequently, the ATLAS ACS–TIMI 51 (Anti-Xa Therapy to Lower Cardiovascular Events in Addition to Standard Therapy in Subjects with Acute Coronary Syndrome–Thrombolysis in Myocardial Infarction-51) trial aimed to test rivaroxaban, a direct and selective inhibitor of factor Xa, in patients with recent ACS (median of 4.7 days) at two different dose regimens, added to standard antithrombotic therapy (low dose ASA plus clopidogrel) [20]. Rivaroxaban 2.5 mg significantly reduced the primary efficacy end point (HR 0.84, 95% CI 0.74–0.96; *p* = 0.008) compared to ASA, with a significant reduction in death from any cause and CV death. However, an increase in major bleedings (2.1% vs. 0.6%; *p* < 0.001), ICH (0.6% vs. 0.2%; *p* = 0.009), and non-major bleedings was observed for both doses over placebo. In the small group of patients with a history of CVD (2% of the total population), no difference was observed in the rate of primary efficacy end point between rivaroxaban and placebo [20], suggesting the augmented risk given by a previous CVD was too high to benefit from a more aggressive antithrombotic regimen. 

Similarly, the APPRAISE-2 (Apixaban for Prevention of Acute Ischemic Events-2) trial tested apixaban, another oral, direct, factor Xa inhibitor, in a population of patients with recent ACS [21]. A twice-daily 5 mg apixaban resulted in an increased incidence of major hemorrhagic episodes without a significant reduction in recurrent CV event, failing to produce a clinical benefit compared to placebo [21]. 

### 5.3. Antiplatelet Strategies for Secondary Prevention

Low-dose ASA is currently recommended as secondary prevention treatment in patients with history of MI or myocardial revascularization [11]. In the secondary prevention trials, ASA yielded an absolute decrease in serious vascular events, specifically with a reduction of about a fifth in total stroke and coronary events [22]. 

Nevertheless, 5–10% of post-MI patients continue to present recurrent CV events despite the use of effective secondary prevention strategies [23]. In this regard, different trials have been conducted to test various antithrombotic regimens as alternatives to ASA alone for long-term CV prevention (Table 2). The CAPRIE (Clopidogrel versus Aspirin in Patients at risk of Ischaemic Events) trial firstly compared clopidogrel to high-dose ASA (325 mg once daily) in a large group of patients with prior MI, stroke, or PAD. In this heterogeneous population, clopidogrel provided an additional 8.7% relative-risk reduction over and above the 25% reduction accepted to be provided by ASA [7]. Considering the subset of patients with symptomatic atherosclerotic disease (prior MI or stroke) enrolled in the trial, the benefit of clopidogrel over ASA was even amplified [24]. Indeed, the 3-year event rate of MACE in these patients was 20.4% with clopidogrel versus 23.8% with ASA, compared with 14.1% in the clopidogrel arm versus 15.2% in the ASA arm in the overall population [24]. Therefore, data identify patients with prior stroke or MI as a high-risk population for recurrent ischemic events, which can benefit more from a more aggressive antiplatelet regimen. 

In the CREDO (Clopidogrel for the Reduction of Events During Observation) trial, a DAPT strategy with clopidogrel added to low-dose ASA was compared with ASA alone in patients undergoing elective PCI for 1 year (6% with a history of CVD) [25]. The combination therapy resulted in a 26.9% relative reduction in the composite risk of death, MI, or stroke, with a non-significant increase in major bleeding (*p* = 0.07) [25]. 

The CHARISMA (Clopidogrel for High Atherothrombotic Risk and Ischemic Stabilization, Management, and Avoidance) trial tested the hypothesis that long-term treatment with a combination of clopidogrel plus ASA might provide a greater protection against CV events than ASA alone in a population of patients with multiple atherothrombotic risk factors or documented CV disease [26]. However, the combination therapy did not significantly reduce the primary endpoint but increased the risk of moderate-to-severe bleeding [26]. Notably, in a post-hoc analysis on patients with prior MI, stroke, or symptomatic PAD, DAPT significantly reduced the rate of primary endpoint (7.3% versus 8.8%; *p* = 0.01), with no significant difference in the rate of severe bleeding (1.7% versus 1.5%, *p* = 0.50) [27]. Therefore, when adopted as secondary prevention regimen in patients with symptomatic CV disease, the association of clopidogrel to ASA seems to produce a significant benefit in terms of reduction of ischemic risk without major bleeding concerns [27].

Ticagrelor has been tested in the PEGASUS-TIMI 54 (Prevention of Cardiovascular Events in Patients with Prior Heart Attack Using Ticagrelor Compared to Placebo on a Background of Aspirin-Thrombolysis in Myocardial Infarction-54) trial as long-term secondary prevention in patients at 1–3 years from a MI [28]. At a median of 33 months, ticagrelor, at two different dose regimens (60 mg twice daily and 90 mg twice daily) on top of low-dose ASA, reduced the incidence of the primary efficacy endpoint (HR for 90 mg of ticagrelor vs. placebo 0.85; *p* = 0.008; HR for 60 mg of ticagrelor vs. placebo 0.84, *p* = 0.004) [28]. As expected, a significant TIMI major bleeding increase was registered (*p* < 0.001 for each dose vs. placebo), though the rate of bleeding leading to severe or irreversible harm was less than 1% over the study period. Nevertheless, it is important to note that population selected for this study presented a low-bleeding risk profile: among others, patients were ineligible in case of prior ischemic stroke or ICH [28]. Therefore, the long-term use of ticagrelor, at the dose of 60 mg twice daily, a more attractive benefit–risk profile dose for the numerically lower rates of bleeding and dyspnea, may be considered in high ischemic and low bleeding risk patients, but it is not recommended in those with a history of previous CVD [11]. 

The TRA2P-TIMI 50 (Thrombin Receptor Antagonist in Secondary Prevention of Atherothrombotic Ischemic Events–Thrombolysis in Myocardial Infarction) trial evaluated vorapaxar, a competitive and selective antagonist of PAR-1, on top of standard antithrombotic therapy as secondary prevention for patients with established atherosclerosis (history of MI, stroke or PAD) [29]. Although vorapaxar reduced the rate of primary ischemic composite endpoint, it was also associated with a significant increase in bleeding, including ICH, particularly heightened in patients with prior stroke. Indeed, the rate of ICH among patients with CVD history was more than doubled by vorapaxar compared to placebo (2.4% versus 0.9%, *p* < 0.001; HR 2.55, 95% CI 1.52–4.28) [29]. In the TRACER (Thrombin Receptor Antagonist for Clinical Event Reduction in Acute Coronary Syndrome) trial, vorapaxar was added to ASA and clopidogrel in ACS patients [30], resulting in an increased risk of major bleeding, including ICH. In a subgroup analysis, no significant interaction was found for both ischemic (*p* = 0.795) and safety (*p* = 0.771) endpoints in the groups with or without prior stroke, despite a trend towards an increased risk of bleeding in patients with a history of stroke (4% of the study population) was observed. Notably, the absolute rate of ICH among patients without a history of stroke was substantially lower (0.2%/year) than patients with prior stroke (0.8%/year) [30]. The Safety Monitoring Board has prematurely interrupted both trials, because of the excessive risk of ICH in an interim analysis. Food and Drug Administration and the European Medicines Agency have designated history of stroke or TIA as a contraindication to vorapaxar use.

### 5.4. Anticoagulant Therapy in Secondary Prevention 

Regarding anticoagulants, vitamin K antagonists, when used in patients with stable CV disease, showed a reduction in the risk of subsequent CV events, countered by a significant increase in bleeding, including ICH [31], and for this reason they are not commonly used in this contest. The COMPASS (Cardiovascular Outcomes for People Using Anticoagulation Strategies) trial randomly assigned 27.395 participants with stable CAD to receive rivaroxaban (2.5 mg twice daily) plus ASA, rivaroxaban (5 mg twice daily), or ASA (100 mg once daily) [32]. The study was early stopped for superiority of the rivaroxaban/ASA arm, which reported a 24% reduction in the primary outcome compared with ASA alone, and a 20% reduction in the risk of the composite net clinical benefit. Despite an increase in major bleeding by 70% (3.1% vs. 1.9%), there was no significant difference in ICH or fatal bleeding. Noteworthily, the association regimen reached a reduction in death from any cause and CV death (HR 0.78, 95% CI 0.64–0.96, *p* = 0.02). In the group of 1032 patients with a history of stroke, the benefit of the combination therapy was consistent with the overall trial results [32]. In a post hoc analysis assessing the incidence of recurrent stroke, rivaroxaban plus ASA resulted in a lower general rate of stroke (HR 0.58; 95% CI 0.44–0.76; *p* < 0.0001), with ischemic stroke reduced by nearly half, as the occurrence of fatal and disabling stroke [33]. Hence, rivaroxaban 2.5 mg may be considered in secondary prevention on top of low-dose ASA, also in prior CVD patients, which may benefit more from a stronger reduction in recurrent ischemic risk. 

## 6. Conclusions

Patients with CAD and CVD represent a relative common subgroup of patients with a challenging antithrombotic management, due to their high ischemic and hemorrhagic risk. Indeed, prior stroke is a marker of frailty and of subsequent risk of hemorrhagic stroke, especially during the first year [34]. For this reason, particular attention must be paid on the choice of antithrombotic strategy, favoring the protection of recurrent ischemic events in the acute phase of myocardial ischemia and focusing on the prevention of bleeding complications at long-term follow-up. In this regard, several ongoing trials are assessing the ideal antithrombotic strategy for this particular subgroup of patients (Table 3).

According to the latest evidence and international guidelines [11,34], prasugrel should be avoided, while ticagrelor, on top of low dose ASA, could be considered in this subgroup of patients presenting with ACS. In case of a recent (<1 year) ischemic stroke, particular attention must be paid, and clopidogrel should be chosen. In the secondary prevention setting, the vascular dose of rivaroxaban added to low dose ASA should be considered, since this strategy demonstrated a significant reduction in the rate of recurrent stroke.

## Figures and Tables

**Table 1:** Major trials on antithrombotic therapies in ACS.

Year	Trial	Population (n)	Prior CVE (%)	Drug	Efficacy End Point	Safety End Point	Efficacy End Point in Stroke Subgroup	Safety end Point in Stroke Subgroup
2001	CURE	12.562	4	Clopidogrel VS Placebo added to ASA	Reduced MACE: 9.3% vs. 11.4%, *p* < 0.001, HR 0.80, 95% CI 0.72–0.90	Increased Major bleeding: 3.7% vs. 2.7%; RR 1.38; *p* = 0.001)		
2007	TRITON-TIMI 38	13.608	4	Prasugrel VS Clopidogre, added to ASA	Reduced MACE: 9.9% vs. 12.1%, HR 0.81, 95% CI: 0.73–0.90; *p* < 0.001;	Increased TIMI Major bleeding non-related to CABG 2.4% VS 1.8% HR 1.32; 95% CI 1.03–1.68; *p* = 0.03. Fatal bleeding (0.4% vs. 0.1%; *p* = 0.002)	Increased MACE: 19.1% VS 14.4%, *p* = 0.15, HR 1.37, 95% CI 0.89–2.13; interaction *p* = 0.02	Increased TIMI major non CABG related bleeding: 5.0% vs. 2.9%, HR 2.46, 95% CI 0.94–6.42; *p* for interaction = 0–22
2009	PLATO	18.624	6	Ticagrelor vs. Clopidogre, added to ASA	Reduced MACE 9.8% vs. 11.7% (HR 0.84; 95% CI 0.77–0.92; *p* < 0.001) CV Death 4.0% vs. 5.1% (HR 0.79, 95% CI 0.69–0.91; *p* = 0.001)	No significant increased PLATO-major bleeding 11.6% vs. 11.2%, HR 1.04, 95% CI 0.95–1.13; *p* = 0.43	No interaction: MACE: 19.0% vs. 20.8% (HR 0.87; 95% CI 0.66–1.13; interaction *p* = 0.84) All-cause Death: 7.9% vs. 13.0% (HR 0.62; 95% CI 0.42–0.91; interaction *p* = 0.49)	No interaction: PLATO major bleeding: 14.6% vs. 14.9% (HR 0.99; 95% CI 0.71–1.37) ICH 0.9% vs. 0.7%, HR 1, 95% CI 0.25–3-99; interaction *p* = 0.38
2011	APPRAISE-2	7.392	10	Apixaban 5 mg VS Placebo	Not reduced MACE: 7.5% vs. 7.9% (HR 0.95; 95% CI 0.80–1.11; *p* = 0.51)	Increased TIMI major bleeding: 1.3% vs. 0.5% (HR 2.59; 95% CI 1.50–4.46; *p* = 0.001)	Trend toward worse outcome (*p* for interaction = 0.08)	No interaction (*p* = 0.31)
2012	TRACER	12.944	4	Vorapaxar VS Placebo, added to standard therapy	Not reduced MACE, recurrent ischemia, urgent coronary revascularization: 18.5% vs. 19.9%; HR 0.92; 95% CI 0.85–1.01; *p* = 0.07	Increased moderate and severe bleeding: 7.2% VS 5.2% HR 1.35; 95% CI 1.16 to 1.58; *p* < 0.001). ICH 1.1% vs. 0.2%, HR 3.39; 95% CI 1.78–6.45; *p* < 0.001	No significant interaction: MACE, recurrent ischemia, urgent coronary revascularization: HR 0.88 (0.63, 1.22), Interaction *p* = 0.795	No significant interaction: Moderate and severe bleeding: 1.48 (0.77, 2.83) interaction *p* = 0.771)
2012	ATLAS ACS -TIMI 51	15.526	3	Rivaroxaban 2.5 VS Rivariaban 5 mg VS placebo	Reduced MACE: 8.9% vs. 10.7% (HR 0.84; 95% CI 0.74–0.96; *p* = 0.008), CV death: 3.3% vs. 4.1%, *p* = 0.04 (HR 0.80, 95% CI 0.65–0.99)	Increased TIMI major bleeding not related to CABG: 2.1% vs. 0.6%, *p* < 0.001; HR 3.96, 95% CI 2.46–6.38). ICH: 0.6% vs. 0.2%, *p* = 0.009	No significant reduction MACE: HR 1.57, 95% CI 0.75–3.31; *p* interaction = 0.1	Four events in rivaroxaban group; none in placebo group (*p* for interaction not possible)
2013	CHAMPION PHOENIX	11.145	5	Cangrelor VS Clopidogrel, added to ASA	Reduced primary end point (death from any cause, MI, ischemia-driven revascularization, stent thrombosis) 4.7% vs. 5.9% (OR 0.78; 95% CI 0.6–0.93; *p* = 0.005)	Not increased severe GUSTO bleeding: 0.16% vs. 0.11% (OR 1.50; 95% CI 0.53 to 4.22; *p* = 0.44)	No interaction *p* = 0.97	No interaction *p* = 0.5

**Table 2:** Major trials on antithrombotic therapies in secondary prevention.

Year	Trial	Population (n)	Setting	Prior CVE (%)	Drug	Efficacy End Point	Safety End Point	Efficacy End Point in Stroke Subgroup	Safety End Point in Stroke Subgroup
1996	CAPRIE	19.185	documented CVD (prior MI, prior stroke, PAD)		Clopidogrel vs. Aspirin	Reduced RR reduction 8.7%, 95% CI 0·3–16·5; *p* = 0·043	Not increased bleedings: 9.27% vs. 9.28%, *p* > 0.05; ICH 0·33% vs. 0·47%, *p* = 0.23	RR reduction 14.9% (95% CI, 0.3–27.3; *p* = 0.045)	Not increased
2002	CREDO	2116	elective PCI	6	Clopidogrel vs. Placebo, added to Aspirin	Reduced RR reduction 27%, 95% CI 3.9–44.4%; *p* = 0.02	Not increased major bleeding (8.8% vs. 6.7%; *p* = 0.07)	Not reported	Not reported
2006	CHARISMA	21.6	multiple CV risk factors or documented CVD	3.245	Clopidogrel vs. Placebo, added to Aspirin	Not increased 6.8% vs. 7.3%, RR 0.93; 95% CI 0.83–1.05; *p* = 0.22	Not significantly increased severe bleeding 1.7% vs. 1.3%; RR 1.25; 95% CI 0.97–1.61 percent; *p* = 0.09. Moderate bleeding 2.1% vs. 1.3% RR 1.62, 95% CI 1.27–2.08; *p* < 0.001	Reduced 7.3% versus 8.8%, *p* = 0.01; HR 0.829, 95% CI 0.719–0.956	Not increased 1.7% versus 1.5%, *p* = 0.50; HR 1.114, 95% CI 0.808–1.535
2015	PEGASUS-TIMI 54	21.162	1 to 3 years prior MI	Excluded	Ticagrelor 60 mg twice vs. Ticagrelor 90 mg twice vs. Placebo, added to Aspirin	Ticagrelor 90 mg vs. placebo: HR 0.85, *p* = 0.008; Ticagrelor 60 mg vs. placebo: HR 0.84, *p* = 0.004	Increased 2.60% with 90 mg vs. 2.30% with 60 mg vs. 1.06% with placebo; *p* < 0.001 for each dose vs. placebo. ICH 0.63%, 0.71%, and 0.60%, respectively	_	_
2019	THEMIS	19.220	Stable CAD and type 2 diabetes mellitus	Excluded	Ticagrelor VS Placebo, added to Aspirin	Reduced MACE 7.7% vs. 8.5%; HR 0.90; 95% CI 0.81–0.99; *p* = 0.04	Increased TIMI major bleeding 2.2% vs. 1.0%; HR 2.32; 95% CI 1.82–2.94; *p* < 0.001	_	_
2017	COMPASS	27.395	Stable CAD	3.8	Rivaroxaban 2.5 mg twice plus ASA, VS Rivaroxaban 5 mg twice plus ASA, VS ASA	Reduced MACE 4.1% vs. 5.4%; HR 0.76; 95% CI 0.66–0.86; *p* < 0.001	Increased major bleeding 3.1% vs. 1.9%; HR 1.70; 95% CI, 1.40–2.05; *p* < 0.001	No interaction	No interaction

**Table 3:** Principal clinical ongoing trials on antithrombotic therapy for CAD patients with history of CVE.

Trial - ClinicalTrials.gov Identifier -	Expected End	Estimated Enrollment	Arms	Aim
PercutaNEOus Coronary Intervention Followed by Monotherapy INstead of Dual Antiplatelet Therapy in the SETting of Acute Coronary Syndromes: The NEO-MINDSET Trial (NEOMINDSET) - NCT04360720 -	August 2023	3400 patients	T alone or P alone for 12 months vs. ASA + T or ASA + P for 12 months	Non-inferiority for ischemic events (Composite of all-cause mortality, CVE, MI or urgent TVR) and superiority for bleeding (BARC ≥2) P2Y12 R inhibitors monotherapy as compared with conventional DAPT in ACS with PCI at 12 months
TAILored Versus COnventional AntithRombotic StratEgy IntenDed for Complex HIgh-Risk PCI (TAILORED-CHIP) - NCT03465644-	December 2023	2000 patients	Low-dose (60 mg) T + ASA for 6 months followed by C alone for 6 months vs. C + ASA for 12 months	Efficacy and safety (net clinical outcome of all-cause death, MI, stroke, stent thrombosis, urgent revascularization, bleeding BARC ≥ 2 at 12 months post-PCI) of early (<6-month post-PCI) intensified and late (>6-month post-PCI) deescalated DAPT in high-risk complex PCI as compared with standard DAPT
LOwer Maintenance Dose TICagrelor in Acute Coronary Syndrome Patients Undergoing Percutaneous Coronary Intervention (LOTIC) - NCT04060914 -	December 2021	225 patients	T 90 mg + ASA for 12 months vs. T 90 mg for 1 month followed by T 60 mg + ASA for 11 months vs. C + ASA for 12 months	T 60 mg after 1 month of standard dose, with antiplatelet activity that is not inferior to the standard dose and better than 75 mg C for patients with ACS after PCI.
SMart Angioplasty Research Team: CHoice of Optimal Anti-Thrombotic Strategy in Patients Undergoing Implantation of Coronary Drug-Eluting Stents 3 (SMART-CHOICE3) - NCT04418479 -	December 2024	5000 patients	C vs. ASA for 1 year after 12 months DAPT	Efficacy (MACCE) and safety of C monotherapy as compared with ASA monotherapy beyond 12 months of standard DAPT after PCI at high risk for recurrent ischemic events.
Ticagrelor Compared to Clopidogrel in Acute Coronary Syndromes (TC4) - NCT04057300 -	March 2021	1500 patients	T + ASA vs. C + ASA	The most effective and safest DAPT regimen (T + ASA or C + ASA) in the North American population of patients presenting with ACS in a cluster randomization design with an electronic health records follow up.

ASA: acetylsalicylic acid; T: ticagrelor; P: prasugrel; C: clopidogrel; DAPT: dual antiplatelet therapy; PCI: percutaneous coronary intervention; DES: drug-eluting stents; ACD: acute coronary syndrome; BARC: Bleeding Academic Research Consortium; MACCE: major adverse cardiovascular and cerebrovascular events; TVR: target vessel revascularization.

## Data Availability

Not applicable.
